# Trends in prevalence and patterns of use of a heated tobacco product ( *IQOS* ^™^) in Japan: A three-year repeated cross-sectional study

**DOI:** 10.12688/f1000research.122491.2

**Published:** 2022-11-21

**Authors:** Karina Fischer, Martha Bajec, Nelly Mainy, Suzana AlMoosawi, Marius Sieverding, Bertram Zwisele, Nathalie Camille, Pierpaolo Magnani, Steve Roulet

**Affiliations:** 1 PMI R&D, Philip Morris Products S.A., Neuchâtel, 2000, Switzerland; 2 Bajec Senseworks Consulting, Hamilton, Ontario, L9A 1L5, Canada; 3 ARGUS - Statistik und Informationssysteme in Umwelt und Gesundheit GmbH, Berlin, 10785, Germany

**Keywords:** Tobacco harm reduction, heated tobacco, smoke-free, IQOS, use patterns, prevalence, smoking initiation, smoking reinitiation

## Abstract

**Background:** Numerous smoke-free tobacco or nicotine-containing product (TNP) alternatives have been introduced to support individual- and population-level harm reduction relative to continued cigarette smoking. This article details the nationwide prevalence and patterns of TNP use between 2016 and 2019 in Japan following the commercialization of *IQOS*™, a smoke-free heated tobacco product (HTP).

**Methods:** Cross-sectional surveys were conducted over a period of three study years (2016/2017, 2017/2018, and 2018/2019) in representative samples of the Japanese general adult population and samples of Japanese adult *IQOS* users registered in the *IQOS* owner database of Philip Morris International’s affiliate in Japan.

**Results:** Across the three study years (Y1-Y3), the prevalence of overall current TNP use (Y1-Y3: 18.5%, 18.9%, and 18.2%) and overall TNP use by age and sex remained similar. However, there was a growing shift from cigarette smoking to smoke-free TNP use across the three study years. While the cigarette smoking prevalence (Y1-Y3: 17.6%, 17.3%, and 16.0%) decreased, the use prevalence of smoke-free TNPs, including the HTP *IQOS*™ (Y1-Y3: 1.8%, 3.2%, and 3.3%) and e-cigarettes (Y1-Y3, 0.7%, 1.6%, and 2.0%) increased. At the same time, TNP initiation, TNP relapse, and TNP reinitiation with *IQOS* were all very low across the three study years. Across Y1-Y3, exclusive use of only one type of TNP (Y1-Y3: 82.3%, 75.0%, and 70.4%) decreased, while dual use of two types of TNPs (Y1-Y3: 14.3%, 17.2%, and 16.7%) increased, and poly-TNP use (Y1-Y3: 2.1%, 6.1%, and 10.0%) increased markedly. Moreover, the majority of adult *IQOS* users were exclusive *IQOS* users.

**Conclusions:** These trends in *IQOS* use behavior suggest that *IQOS*™ has the potential to switch adult smokers from cigarettes to smoke-free tobacco products, which presents a harm reduction opportunity, and that HTPs are effective tools for complementing current tobacco control measures.

## Introduction

It is well established that cigarette smoking can lead to numerous negative health outcomes, including premature and preventable death ^1^. The burden of smoking on individual and population health has driven health authorities and regulatory bodies to recommend and implement various tobacco control policies ^2^. Never initiating or quitting smoking are the most direct ways to alleviate the health burden of smoking ^3^. However, strategies aimed at preventing smoking and promoting cessation continue to face numerous challenges, including smokers who are not motivated to quit or who relapse/reinitiate smoking after a period of abstinence ^2, 4, 5^. While smoking prevalence has declined over the last decades, over 1 billion people globally continue to smoke combustible tobacco products today ^1, 6^, and cigarette smoking continues to be responsible for the largest number of preventable deaths worldwide ^7, 8^.

To complement tobacco control efforts ^9^, tobacco harm reduction strategies have been introduced around the world ^10, 11^. Tobacco harm reduction includes prevention of tobacco or nicotine-containing product (TNP) use initiation and reinitiation ^2, 11, 12^ while ensuring that adult smokers switch completely from combustible TNPs to less harmful smoke-free (i.e., non-combustible) TNPs ^13, 14^.

Unlike cigarettes, which burn tobacco and produce a complex mixture of harmful and potentially harmful constituents (HPHC) through combustion, *IQOS*™, a smoke-free heated tobacco product (HTP) developed by Philip Morris International (PMI), heats a specifically engineered tobacco stick (i.e., *HEETS*™/ *HeatSticks*™) to temperatures below the level of combustion ^15^. As a consequence, smokers who switch completely to *IQOS* use are exposed to much lower levels of HPHCs than those who continue smoking cigarettes ^16– 20^.

As part of PMI’s commitment to a smoke-free future, *IQOS*™ was introduced in Japan in 2014 and is now available in more than 70 countries worldwide, with an estimated 21 million adult users globally ^21^. The availability and demand for *IQOS* as an alternative to cigarettes has raised the need to monitor *IQOS* use prevalence and use patterns with the aim of informing public health authorities locally and worldwide. Such findings will further enable regulators to delineate the role of *IQOS* in harm reduction as a viable substitute for cigarettes ^2, 22^.

Building on the reporting of Afolalu *et al.* ^23^, the aim of the current study was to analyze the temporal trends in TNP use in nationally representative samples of the Japanese general adult population (JGAP) and, separately, in samples of Japanese adult *IQOS* users (JA *IQOS*) from PMI’s adult *IQOS* owner database in Japan across three recent years (2016/2017, 2017/2018, and 2018/2019).

## Participants and methods

### Setting

**Figure 1.**
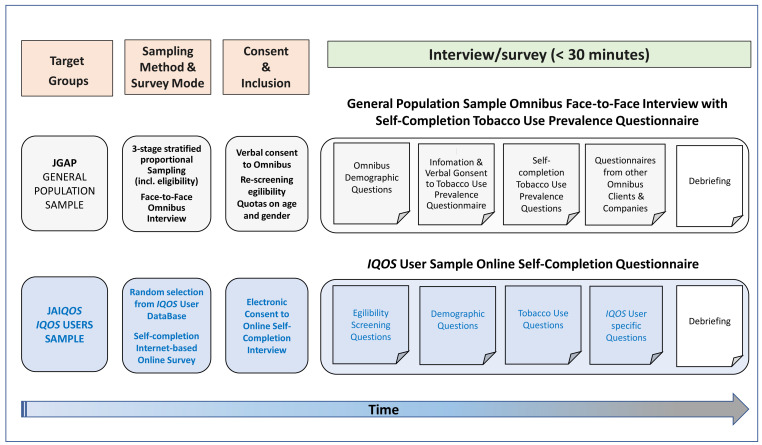
Overall survey study design for the JGAP and JA *IQOS* samples in each wave of the 3-year study period (2016/2017, 2017/2018, and 2018/2019) of the survey fielding. Abbreviations: ICF, informed consent form; JA *IQOS*, sample of adult Japanese *IQOS* users from PMI’s *IQOS*™ owner database in Japan; JGAP, representative sample of the Japanese general adult population; PMI, Philip Morris International.

Cross-sectional surveys in representative samples of the Japanese general adult population (JGAP) and, separately, in samples of Japanese adult *IQOS* users (JA *IQOS*) registered in the *IQOS* owner database of Philip Morris International (PMI)’s affiliate in Japan were initiated in December 2016 and repeated annually over three full calendar years from 2016/2017 to 2018/2019 ( Figure 1). Considering that *IQOS* ^TM^ was relatively new on the Japanese TNP market in 2016, the *IQOS* use prevalence in the JGAP was expected to be low. Therefore, additional surveys among JA *IQOS* samples were conducted alongside the JGAP surveys to obtain reliable estimates of *IQOS* use patterns among Japanese adult *IQOS* users ^23^.

### Participants and study design

**Study participants**

To be included in the JGAP or JA *IQOS* samples, individuals had to be of legal age for purchasing TNPs in Japan (i.e., ≥20 years), current residents of Japan, and fluent in Japanese. Those included in the JA *IQOS* samples also had to have used >100 *HEETS*™ */HeatSticks*™ in their lifetime ^24^, be a current user of *IQOS*™ with *HEETS/HeatSticks*, have access to the internet, and not be currently employed by PMI or its affiliates.

***JGAP — Sampling, sample size, and survey mode***

The JGAP samples were obtained via a syndicated (Omnibus) survey overseen and coordinated by an independent global consumer research company. The fieldwork provider in Japan was Central Research Services Inc (Tokyo, Japan). The Omnibus surveys employed a three-stage stratified proportional random sampling strategy that included the whole country. In stage 1, sampling points in the 12 Japanese administrative regions were allocated on the basis of their share of the population ^25^. Households within each sampling point were identified in Stage 2 by using an electronic residential map, from which about 40 households were randomly selected. In the final stage 3, participants who met the inclusion criteria were selected from within the sampled households. Within each sampling point, quotas on age and sex were set to ensure the representativeness of the Japanese population.

**Figure 2.**
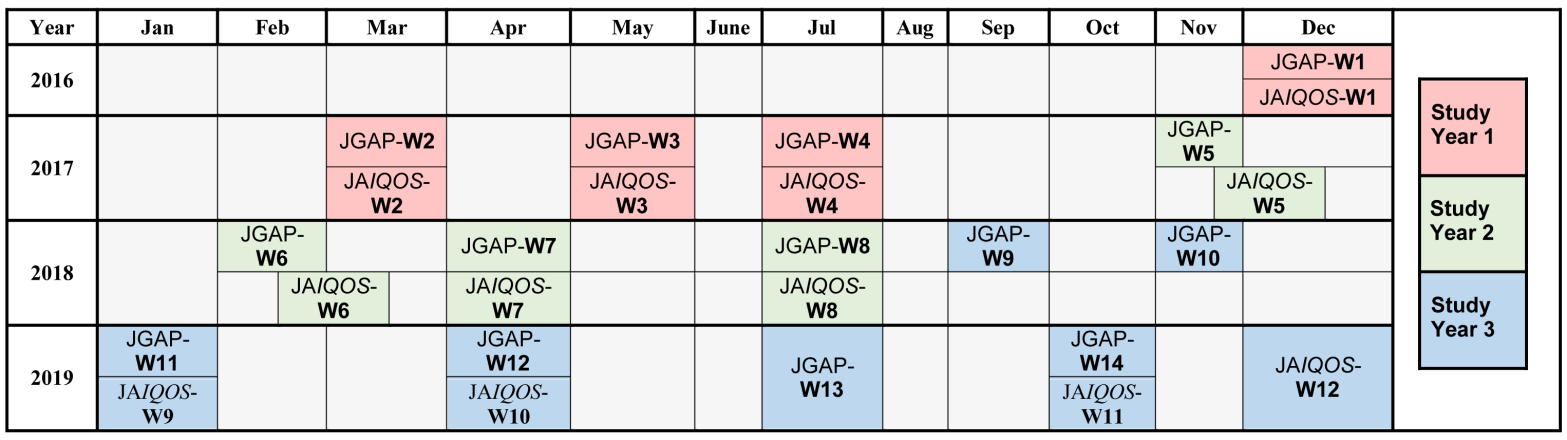
Allocation of the JGAP and JA *IQOS* survey waves over the 3-year study period (2016/2017, 2017/2018, and 2018/2019). Abbreviations: JA *IQOS*, sample of adult Japanese *IQOS*™ users from PMI’s *IQOS* owner database in Japan; JGAP, representative sample of the Japanese general adult population; PMI, Philip Morris International; W, survey wave.

The annual JGAP sampling consisted of four approximately equal-sized waves spaced throughout each study year to account for potential seasonal differences ( Figure 2). A sample size of 5,000 participants per year was sufficient to estimate an *IQOS*™ use prevalence of 5.0% with 95% confidence and a precision of ±0.6% units. In the third year, six survey waves (7,000 participants) were conducted to increase the sample size and improve the accuracy of the estimates.

The JGAP surveys were conducted at participants’ homes through in-person face-to-face pen-and-paper interviews. However, to avoid any bias on basis of social desirability of their response regarding their personal TNP use, the participants were handed the “Tobacco Use Prevalence” questionnaire section for self-completion. For completing the Omnibus questionnaires, each participant was given a coupon for JPY 500 (approximately USD 4).

**JA *IQOS — Sampling, sample size, and survey mode***

Upon purchasing an *IQOS*™ device, users were invited to register in the PMI Japan *IQOS* owner database, which included about 350,000 adult *IQOS* owners in July 2017 and reached close to six million in 2019. Considering the demographic age-sex distribution of the database, individuals were randomly selected from the database and invited by email to participate in the survey for each wave.

A sample size of 2,000 participants per year was sufficient to estimate a 50% proportion of exclusive *IQOS*™ use with 95% confidence and a precision of ±2.19% units. Each annual *IQOS* user sample consisted of four approximately equal-sized waves spaced throughout each study year to account for potential seasonal differences ( Figure 2), with the aim of recruiting 500 adult participants per wave. The JA *IQOS* surveys were conducted entirely online through computer-assisted self-interviewing. For completing the online survey, participants were given a gift code valued at JPY 500 (about USD 4). The existing standard TNP use questions are available in the literature.

**Survey questionnaires**

For the present study, the “Tobacco Use Prevalence” questionnaire was developed on the basis of several existing standard TNP use questions available in the literature to capture information about TNP use such as the (i) CDC Adult Tobacco Use Questions of the National Health Interview Survey (NHIS), (ii) WHO/CDC/CPHA Questions of the Global Adult Tobacco Survey (GATS), (iii) NIH/FDA PATH Study questionnaires, and (iv) MHLW Tobacco Use Questions of the Japan National Health and Nutrition Survey. The questionnaire was not specifically validated. In both the JGAP and JA *IQOS* samples, the same questionnaire was used. However, while for the JGAP samples a pen-and-paper self-completion survey mode was used, for the JA *IQOS* samples an online mode was applied. The survey questions can be found in the Extended data.

**Ethical conduct of the study**

All subjects gave informed consent for inclusion in the study prior to participation. The participants of the JGAP samples gave verbal consent that was recorded by the interviewers as part of the Omnibus interviews, while the participants of the JA *IQOS* samples provided written consent. The study was conducted in accordance with the ethical principles that have their origin in the Declaration of Helsinki and were consistent with Good Epidemiological Practice (GEP) ^26^. The study protocol, including the procedures of providing informed consent, were approved by the Hakata Clinic Institutional Review Board (Reference ID: J-186) in Fukuoka, Japan. 

**Analytical methods**

Analyses were conducted using SAS v9.4 (or higher; SAS Inc., Cary, North Carolina, USA). For both the JGAP and JA *IQOS* samples, data were analyzed and summarized descriptively for each study year. For participant characteristics and outcome measures, continuous data are presented as mean and standard deviation (SD) or 95% confidence intervals (CI) and categorical data as number and percentage (95% CI) for the total samples and/or stratified by age and sex. Missing data were not included in the statistical analyses.

The following definitions were applied: “Use/never use” of cigarettes or *IQOS*™ with *HEETS*™ */HeatSticks*™: having/not having used 100 cigarettes or 100 *HEETS/HeatSticks* in the lifetime, to differentiate established cigarette or *IQOS* users from triers or experimenters ^24^. “Current use”: daily or nondaily use of a TNP at the time of the survey. “Exclusive”, “dual”, and “poly” use: current use of only one type, two types, or three or more types of TNPs, respectively. “Initiation”: the time point at which a participant started established use/smoking of a TNP. “Initiation rate”: proportion of initiation in the last 12 months among never TNP users. “Relapse” and “reinitiation”: restarting TNP use following a period of quitting all TNPs for ≤12 months and >12 months, respectively.

Prevalence of current TNP use for overall TNPs or by TNP category (cigarettes, *IQOS*™, e-cigarettes, etc.) was calculated in the JGAP samples. For both JGAP and JA *IQOS* samples, the following was calculated: response rates, sample characteristics, and patterns of TNP use (JGAP: exclusive, dual, and poly use; JA *IQOS*: exclusive *IQOS* use and *IQOS* use with combustible or smoke-free TNP) as well as frequency (past 30-day use), intensity (average daily consumption), and history (JGAP: initiation, relapse, and reinitiation with *IQOS*; JA *IQOS*: previous cigarette smoking history before starting *IQOS* use) of TNP use.

## Results

### Survey dispositions and outcome rates

**Figure 3.**
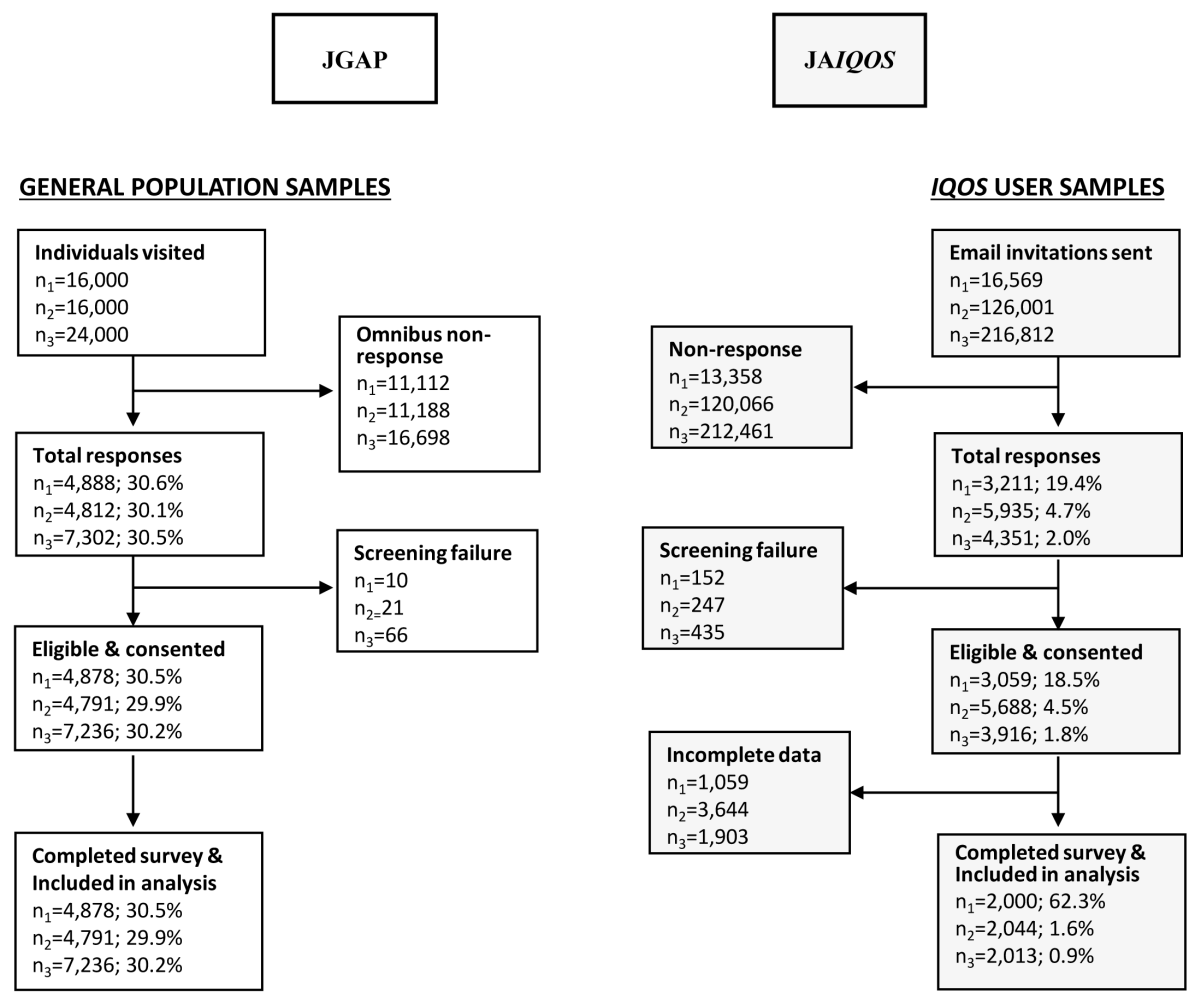
Flow diagram of study samples and survey dispositions in the JGAP and JA *IQOS* samples. Abbreviations: JA *IQOS*, sample of adult Japanese *IQOS*™ users from PMI’s *IQOS* owner database in Japan; JGAP, representative sample of the Japanese general adult population; n1-n3, sample sizes for study years 1–3, respectively; PMI, Philip Morris International.

Regarding survey dispositions and outcome rates ( Figure 3), the JGAP samples had a response rate of >30% in each of the three study years (Y1-Y3), which resulted in sample sizes of 4,878, 4,791, and 7,236 for Y1-Y3 of the Omnibus survey, respectively. In the Y1-Y3 JA *IQOS* samples, response rates of 19.4%, 4.7%, and 2.0% yielded sample sizes of 2,000, 2,044, and 2,013, respectively. 

**Sample characteristics**

***JGAP samples***

Overall, the demographic characteristics of the JGAP samples were similar across Y1-Y3 ( Table 1) and comparable with those of the Japanese adult population ^25^. The mean (±SD) ages of the Y1-Y3 samples were 53.8 (±17.9), 54.5 (±17.6), and 54.8 (±17.8) years, respectively, and each of the samples included slightly more women (Y1-Y3: 51.9%, 53.3% and 53.2%) than men (Y1-Y3: 48.1%, 46.7%, and 46.8%), mirroring the female skew in the actual Japanese population ^25^.

**Table 1.** Sample characteristics of the JGAP and JA *IQOS* samples.

	Japanese population (2016) * (%)	Number (n) and percentage (% [95% CI])					
		Year 1 (2016/2017)		Year 2 (2017/2018)		Year 3 (2018/2019)	
		JGAP (N=4,878)	JA *IQOS* (N=2,000)	JGAP (N=4,791)	JA *IQOS* (N=2,044)	JGAP (N=7,236)	JA *IQOS* (N=2,013)
Sex							
Men	48.3	2,345 48.1 [46.6–49.5]	1,632 81.6 [79.8–83.3]	2,238 46.7 [45.2–48.2]	1,641 80.3 [78.4–82.0]	3,385 46.8 [45.6–48.0]	1,609 79.9 [78.1–81.7]
Women	51.7	2,533 51.9 [50.5–53.4]	368 18.4 [16.7–20.2]	2,553 53.3 [51.8–54.8]	403 19.7 [18.0–21.6]	3,851 53.2; [52.0–54.4]	404 20.1 [18.3–21.9]
Age (years)							
20–29	12	528 10.8 [9.9–11. 8]	420 21.0 [19.2–22.9]	464 9.7 [8.8–10.6]	346 16.9 [15.3–18.7]	704 9.7 [9–10.5]	330 16.4 [14.8–18.1]
30–39	15.1	723 14.8 [13.8–15.9]	736 36.8 [34.6–39.0]	668 13.9 [12.9–15.0]	710 34.7 [32.6–36.9]	978 13.5 [12.7–14.4]	710 35.3 [33.1–37.5]
40–49	17.8	873 17.9 [16.8–19.1]	568 28.4 [26.4–30.5]	886 18.5 [17.4–19.7]	642 31.4 [29.4–35.5]	1,336 18.5 [17.5–19.4]	644 32.0 [29.9–34.1]
50+	55.2	2,754 56.5 [55.0–57.9]	276 13.8 [12.3–15.4]	2,773 57.9 [56.4–59.3]	346 16.9 [15.3–18.7]	4,218 58.3 [57.1–59.5]	329 16.3 [14.7–18.1]
Mean [±SD]	-	53.8 [±17.9]	38.5 [±9.7]	54.5 [±17.6]	39.7 [±10.1]	54.8 [±17.8]	39.9 [±9.9]
Education							
Junior high school	8.6	454 9.3 [8.5–10.2]	124 6.2 [5.1–7.4]	417 8.7 [7.9–9.6]	139 6.8 [5.7–8.0]	619 8.6 [7.9–9.3]	151 7.5 [6.3–8.8]
High school	40.1	2,395 49.1 [47.6–50.6]	726 36.3 [34.1–38.5]	2,433 50.8 [49.3–52.3]	753 36.8 [34.7–39.0]	3,603 49.8 [48.6–51]	744 37.0 [34.8–39.2]
College/University	41.8	1,980 40.6 [39.2–42.0]	1,135 56.8 [54.5–59.0]	1,917 40.0 [38.6–41.5]	1,114 54.4 [52.3–56.7]	2,967 41.0 [39.8–42.2]	1,085 53.9 [51.6–56.1]
Don’t know/NA	9.5	49 1.0 [0.7–1.4]	15 0.8 [0.4–1.3]	24 0.5 [0.3–0.8]	38 1.9 [1.3–2.6]	47 0.6 [0.4–0.9]	33 1.6 [1.1–2.3]
Occupation							
Farming/Agriculture/Fishery	-	80 1.6 [1.3–2.1]	8 0.4 [0.1–0.8]	89 1.9 [1.4–2.3]	15 0.7 [0.4–1.3]	165 2.3 [1.9–2.7]	18 0.9 [0.5–1.5]
Self-employed/Small private business	12.1	538 11.0 [10.1–12.0]	329 16.5 [14.8–18.2]	511 10.7 [9.8–11.6]	347 17.0 [15.3–18.7]	848 11.7 [10.9–12.5]	354 17.6 [15.9–19.4]
Clerical employee	-	927 19.0 [17.9–20.2]	284 14.2 [12.6–15.9]	845 17.6 [16.5–18.8]	228 11.2 [9.8–12.6]	1,289 17.8 [16.9–18.8]	271 13.5 [12–19.4]
Manual employee	-	1,063 21.8 [20.6–23.0]	268 13.4 [11.9–15.0]	1,094 22.8 [21.6–24.1]	272 13.3 [11.8–14.9]	1,560 21.6 [20.6–22.6]	253 12.6 [11.1–14.1]
Managing profession	46.9	118 2.4 [2.0–2.9]	414 20.7 [18.9–22.6]	108 2.3 [1.8–2.8]	432 21.1 [19.3–23.0]	197 2.7 [2.3–3.2]	398 19.8 [18.0–21.6]
Housewife	19.9	1,211 24.8 [23.6–26.1]	84 4.2 [3.3–5.2]	1,175 24.5 [23.3–25.8]	115 5.6 [4.6–6.8]	1,791 24.8 [23.7–25.8]	95 4.7 [3.8–5.8]
Student	2	106 2.2 [1.7–2.7]	37 1.9 [1.3–2.6]	82 1.7 [1.3–2.2]	34 1.7 [1.1–2.4]	160 2.2 [1.8–2.6]	28 1.4 [0.9–2.1]
Retired/Unemployed	19.1	835 17.1 [16.0–18.3]	26 1.3 [0.8–1.9]	887 18.5 [17.3–19.7]	69 3.4 [2.6–4.3]	1,226 16.9 [16–17.9]	43 2.1 [1.5–2.9]
Don’t know/NA	-	NA	550 27.5 [25.5–29.6]	NA	532 26.0 [24.1–28.0]	NA	553 27.5 [25.5–29.5]
Region							
Chubu	-	964 19.8 [18.6–21.0]	310 15.5 [13.9–17.2]	908 19.0 [17.8–20.1]	288 14.1 [12.6–15.7]	964 19.8 [18.6–21.0]	285 14.2 [12.6–15.8]
Chugoku	-	294 6.0 [5.3–6.8]	94 4.7 [3.8–5.8]	281 5.9 [5.2–6.6]	95 4.6 [3.7–5.7]	294 6.0 [5.3–6.8]	87 4.3 [3.4–5.4]
Hokkaido	-	219 4.5 [3.9–5.2]	58 2.9 [2.2–3.8]	214 4.5 [3.8–5.1]	93 4.5 [3.6–5.6]	219 4.5 [3.9–5.2]	93 4.6 [3.7–5.7]
Kanto	-	1,585 32.5 [31.1–33.9]	871 43.6 [41.3–45.8]	1,610 33.6 [32.2–35.0]	827 40.5 [38.3–42.7]	1,585 32.5 [31.1–33.9]	833 41.4 [39.2–43.6]
Kinki	-	757 15.5 [14.5–16.6]	342 17.1 [15.4–18.9]	728 15.2 [14.1–16.3]	349 17.7 [15.4–18.8]	757 15.5 [14.5–16.6]	333 16.5 [14.9–18.3]
Kyusyu	-	549 11.3 [10.3–12.2]	148 7.4 [6.2–8.7]	553 11.5 [10.6–12.5]	190 9.3 [8.0–10.7]	549 11.3 [10.3–12.2]	177 8.8 [7.5–10.2]
Shikoku	-	161 3.3 [2.8–3.9]	41 2.1 [1.4–2.8]	142 3.0 [2.5–3.5]	38 1.9 [1.3–2.6]	161 3.3 [2.8–3.9]	37 1.8 [1.2–2.6]
Tohoku	-	349 7.2 [6.4–8.0]	136 6.8 [5.7–8.0]	355 7.4 [6.6–8.2]	164 8.0 [6.8–9.3]	349 7.2 [6.4–8.0]	168 8.3 [7.1–9.7]

Abbreviations: CI, confidence interval; JA *IQOS*, sample of adult Japanese *IQOS* users from PMI’s *IQOS* owner database in Japan; JGAP, representative sample of the Japanese general adult population; NA, not applicable; PMI, Philip Morris International; SD, standard deviation. *Source: Statistics Bureau of Japan (2015) Source on Education: Statistics Bureau of Japan (2010) Source on Occupation: Public Opinion Survey on the Life of the People (23 June - 10 July 2016).

In each of Y1-Y3, a larger proportion of the sample was based in a major city (Y1-Y3: 27.4%, 28.1%, and 28.6%) than in rural areas (Y1-Y3: 10.0%, 9.3%, and 8.8%). Across Y1-Y3 ( Table 1), most of the samples reported high school (Y1-Y3: 49.1%, 50.8%, and 49.8%) or college/university (Y1-Y3: 40.6%, 40.0%, and 41.0%) as the highest level of education, and the most common occupations were homemaker (Y1-Y3: 24.8%, 24.5%, and 24.8%), manual employee (Y1-Y3: 21.8%, 22.8%, and 21.6%), and clerical employee (Y1-Y3: 19.0%, 17.6%, and 17.8%).

**JA *IQOS samples***

Overall, the demographic characteristics of the JA *IQOS* samples were similar across Y1-Y3 ( Table 1). The mean (±SD) ages of the Y1-Y3 samples were 38.5 (±9.7), 39.7 (±10.1), and 39.9 (±9.9) years, respectively, and in each of the samples there were more men (Y1-Y3: 81.6%, 80.3%, and 79.9%) than women (Y1-Y3: 18.4%, 19.7%, and 20.1%).

Across Y1-Y3 ( Table 1), most of the participants reported completing college/university (Y1-Y3: 56.8%, 54.4%, and 53.9%) or high school (Y1-Y3: 36.3%, 36.8%, and 37.0%), and the most common occupations were manager (Y1-Y3: 20.7%, 21.1%, and 19.8%) and self-employed/small business owner (Y1-Y3: 16.5%, 17.0%, and 17.6%). 

**TNP use in JGAP samples**

***Prevalence of overall TNP use***

Across Y1-Y3, the prevalence of overall current (Y1-Y3: 18.5%, 18.9%, 18.2%) former (Y1-Y3: 18.7%, 16.3%, 16.9%), and never (Y1-Y3: 62.9%, 64.8%, 64.9%) TNP use as well as of TNP use by age and sex were similar ( Table 2).

**Table 2.** Prevalence of overall current, former, and never TNP use in the JGAP samples overall and by sex and age group.

		Number (n) and percentage (% [95% CI])								
		Current TNP users			Former TNP users			Never TNP users		
	Age Group (years)	Year 1 (2016/2017)	Year 2 (2017/2018)	Year 3 (2018/2019)	Year 1 (2016/2017)	Year 2 (2017/2018)	Year 3 (2018/2019)	Year 1 (2016/2017)	Year 2 2017/2018)	Year 3 (2018/2019)
All	All	894 18.5 [17.3–19.6]	900 18.9 [17.7–20.1]	1,304 18.2 [17.2–19.1]	905 18.7 [17.5–19.9]	777 16.3 [15.2–17.4]	1,211 16.9 [16.0–17.8]	3,044 62.9 [61.4–64.3]	3,086 64.8 [63.4–66.2]	4,656 64.9 [63.8–66.1]
	20–29	110 20.8 [17.4–24.6]	84 18.1 [14.7–22]	129 18.4 [17.2–19.1]	30 5.7 [3.8–8.1]	25 5.4 [3.5–7.9]	40 5.7 [4.1–7.8]	388 73.5 [69.5–77.3]	354 76.5 [72.3–80.3]	531 75.9 [72.5–79]
	30–39	180 25.0 [21.8–28.4]	173 26.0 [22.6–29.5]	244 25.2 [15.6–21.6]	115 16.0 [13.3–18.9]	92 13.8 [11.2–16.7]	143 14.8 [12.5–17.2]	425 59.0 [55.3–62.7]	401 60.2 [56.3–64]	581 60.0 [56.8–63.2]
	40–49	222 25.5 [22.6–28.6]	213 24.2 [21.3–27.2]	314 23.7 [22.4–28.1]	145 16.7 [14.2–19.4]	136 15.4 [13.1–18]	200 15.1 [13.2–17.2]	502 57.8 [54.4–61.1]	532 60.4 [57–63.7]	809 61.1 [58.4–63.8]
	50+	382 14.0 [12.7–15.4]	430 15.6 [14.2–17.1]	617 14.8 [13.6–15.9]	615 22.6 [21–24.2]	524 19.0 [17.5–20.6]	828 19.8 [18.6–21.1]	1729 63.4 [61.5–65.3]	1,799 65.3 [63.5–67.2]	2,735 65.4 [63.9–66.9]
Men	All	683 29.4 [27.5–31.4]	686 30.9 [29–33]	994 29.7 [28.1–31.3]	723 31.1 [29.2–33.1]	620 28.0 [26.1–29.9]	965 28.8 [27.2–30.4]	916 39.4 [37.4–41.5]	911 41.1 [39–43.2]	1,389 41.5 [39.8–43.2]
	20–29	74 27.6 [22.3–33.4]	62 27.7 [21.9–34.1]	86 24.0 [19.6–28.8]	19 7.1 [4.3–10.9]	12 5.4 [2.7–9.2]	24 6.7 [4.3–9.9]	175 65.3 [59.2–71]	150 67.0 [60.3–73.1]	248 69.3 [64.2–74.1]
	30–39	142 36.0 [31.2–41]	139 40.3 [35–45.7]	189 37.3 [33–41.7]	68 17.3 [13.6–21.4]	55 15.9 [12.2–20.3]	90 17.8 [14.5–21.4]	184 46.7 [41.6–51.8]	151 43.8 [38.4–49.2]	228 45.0 [40.5–49.5]
	40–49	162 38.9 [34.2–43.9]	155 37.0 [32.3–41.9]	241 37.4 [33.6–41.3]	102 24.5 [20.4–29]	96 22.9 [18.9–27.3]	143 22.2 [19–25.7]	152 36.5 [31.9–41.4]	168 40.1 [35.3–45]	260 40.4 [36.5–44.3]
	50+	305 24.5 [22.1–27.1]	330 26.9 [24.3–29.5]	478 26.0 24.0–28.1]	534 42.9 [40.1–45.8]	457 37.2 [34.4–40]	708 38.5 [36.2–40.8]	405 32.6 [29.9–35.3]	442 36.0 [33.2–38.8]	653 35.5 [33.3–37.8]
Women	All	211 8.4 [7.3–9.6]	214 8.4 [7.3–9.6]	310 8.1 [7.2–9.1]	182 7.2 [6.2–8.4]	157 6.2 [5.2–7.2]	246 6.4 [5.6–7.3]	2,128 84.4 [82.9–85.9]	2175 85.4 [83.9–86.8]	3,267 85.5 [84.2–86.6]
	20–29	36 13.8 [9.8–18.7]	22 9.2 [5.8–13.7]	43 12.6 [9.2–16.6]	11 4.2 [2.1–7.5]	13 5.4 [2.9–9.2]	16 4.7 [2.6–7.5]	213 81.9 [76.6–86.5]	204 85.4 [80.2–89.6]	283 82.7 [78.3–86.7]
	30–39	38 11.7 [8.3–15.7]	34 10.6 [7.4–14.5]	55 11.9 [9.1–15.3]	47 14.4 [10.7–18.8]	37 11.5 [8.2–15.6]	53 11.5 [8.7–14.8]	241 73.9 [68.8–78.7]	250 77.9 [72.9–82.4]	353 76.6 [72.4–80.4]
	40–49	60 13.2 [10.2–16.8]	58 12.6 [9.6–16]	73 10.8 [8.5–13.4]	43 9.5 [6.9–12.6]	40 8.7 [6.2–11.7]	57 8.4 [6.4–10.8]	350 77.3 [73.1–81.1]	364 78.8 [74.7–82.5]	549 80.9 [77.6–83.8]
	50+	77 5.2 [4.1–6.5]	100 6.6 [5.3–8.0]	139 5.9 [5.0–7.0]	81 5.5 [4.3–6.8]	67 4.4 [3.4–5.6]	120 5.1 [4.2–6.1]	1,324 89.3 [87.6–90.9]	1,357 89.0 [87.3–90.6]	2,082 88.9 [87.5–90.2]

Abbreviations: CI, confidence interval; JGAP, representative sample of the Japanese general adult population.

***Prevalence of individual TNP use***

Cigarette smoking prevalence decreased from 17.6% in Y1 to 16.0% in Y3, while the use prevalence of other TNPs, including HTPs and e-cigarettes, increased ( Table 3). The use prevalence of all HTP brands (i.e., *IQOS*™, Ploom/Ploom Tech, and glo) increased across Y1-Y3, and, of all HTP brands surveyed, *IQOS* had the highest use prevalence (Y1-Y3: 1.8%, 3.2%, 3.3%). The use prevalence of e-cigarettes increased from 0.7% to 1.6% to 2.0% during Y1-Y3.

**Table 3.** Prevalence of individual TNP use in the JGAP samples overall and by sex and age group.

		Number (n) and percentage (% [95% CI])		
	Study year *	Cigarettes **	*IQOS*™	E-cigarettes
Overall				
	1	852 17.6 [16.5–18.7]	86 1.8 [1.4–2.2]	35 0.7 [0.5–1.1]
	2	825 17.3 [16.2–18.5]	152 3.2 [2.7–3.8]	76 1.6 [1.2–2.0]
	3	1150 16.0 [15.1–17.0]	240 3.3 [2.9–3.8]	146 2.0 [1.7–2.4]
Sex				
	1	654 28.2 [26.3–30.1]	70 3.0 [2.3–3.8]	25 1.1 [0.6–1.6]
Men	2	630 28.4 [26.5–30.4]	114 5.1 [4.2–6.2]	59 2.7 [2.0–3.5]
	3	882 26.3 [24.8–27.9]	181 5.4 [4.6– 6.3]	104 3.1 [2.5–3.8]
	1	198 7.9 [6.8 *–*9.0]	16 0.6 [0.3–1.1]	10 0.4 [0.1–0.8]
Women	2	195 7.7 [6.6–8.8]	38 1.5 [1.0–2.1]	17 1.1 [0.3–1.1]
	3	268 7.0 [6.2–7.9]	59 1.5 [1.1–2.0]	42 1.1 [0.7–1.5]
Age group (years)				
	1	98 18.6 [15.3 *–*22.2]	20 3.8 [2.3–5.8]	9 1.7 [0.7–3.3]
20 *–*29	2	70 15.1 [11.9–18.8]	23 5.0 [3.1–7.4]	14 3.0 [1.6–5.1]
	3	108 15.4 [12.8–18.4]	37 5.3 [3.7–7.3]	22 3.1 [1.9–4.8]
	1	172 23.9 [20.8 *–*27.2]	23 3.2 [2.0–4.8]	6 0.8 [0.3–1.9]
30–39	2	145 21.8 [18.625.2]	58 8.7 [6.6–11.2]	16 2.4 [1.3–3.9]
	3	196 20.2 [17.7–23.0]	87 9.0 [7.2–11.0]	38 3.9 [2.7–5.4]
	1	214 24.6 [21.7 *–*27.7]	25 2.9 [1.8–4.3]	9 1.0 [0.4–2.0]
40 *–*49	2	196 22.2 [19.5–25.2]	36 4.1 [2.8–5.7]	23 2.6 [1.6–3.9]
	3	270 20.4 [18.2–22.7]	62 4.7 [3.6–6.0]	37 2.8 [1.9–3.9]
	1	368 13.5 [12.2–14.9]	18 0.7 [0.3–1.1]	11 0.4 [0.2–0.8]
50+	2	414 15.0 [13.7–16.5]	35 1.3 [0.8–1.8]	23 0.8 [0.5–1.3]
	3	576 13.8 [12.7–14.9]	54 1.3 [0.9–1.7]	49 1.2 [0.8–1.6]

Abbreviations: CI, confidence interval; JGAP, representative sample of the Japanese general adult population; TNP, tobacco or nicotine-containing product. *Year 1 (2016/2017), Year 2 (2017/2018), and Year 3 (2018/2019) **Cigarettes include hand-rolled cigarettes

In each of Y1-Y3, cigarette smoking was more prevalent among men (Y1-Y3: 28.2%, 28.4%, and 26.3%) than women (Y1-Y3: 7.9%, 7.7%, and 7.0%) and was highest among 40–49-year-olds (Y1-Y3: 24.6%, 22.2%, and 20.4%). The *IQOS*™ use prevalence in each of Y1-Y3 was higher among men (Y1-Y3: 3.0%, 5.1%, and 5.4%) than women (Y1-Y3: 0.6%, 1.5%, and 1.5%) and was highest among 20–29-year-olds (3.8%) in Y1, but shifted to be highest among 30–39-year-olds in Y2 (8.7%) and Y3 (9.0%). In both Y1 (1.7%) and Y2 (3.0%), e-cigarette use prevalence was highest among 20–29-year-olds, but in Y3 shifted to be highest among 30–39-year-olds (3.9%; Table 3).

***Patterns of TNP use***

Across Y1-Y3 ( Table 4), exclusive use of only one type of TNP decreased (Y1-Y3: 82.3%, 75.0%, and 70.4%), while dual use of two types of TNPs increased (Y1-Y3: 14.3%, 17.2%, and 16.7%) and poly-TNP use increased markedly (Y1-Y3: 2.1%, 6.1%, and 10.0%).

**Table 4.** TNP use patterns in the JGAP samples.

	Number (n) and percentage (% [95% CI])		
	Year 1 (2016/2017) (n=887)	Year 2 (2017/2018) (n=900)	Year 3 (2018/2019) (n=1,304)
Exclusive use	730 82.3 [79.6–84.8]	675 75.0 [72.0–77.8]	918 70.4 [67.8–72.9]
Cigarettes *	705 79.5 [76.6–82.1]	613 68.1 [64.9–71.2]	822 63.0 [60.3–65.7]
*IQOS*™	22 2.5 [1.5–3.8]	43 4.8 [3.4–6.4]	69 5.3 [4.1–6.7]
E-cigarettes	3 0.3 [0.0–1.0]	10 1.1 [0.5–2.1]	6 0.5 [0.1–1.0]
One other TNP	-	9 1.0 [0.4–1.9]	21 1.6 [0.9–2.5]
Dual use	127 14.3 [12.0–16.8]	155 17.2 [14.8–19.9]	218 16.7 [14.7–18.9]
Cigarettes + other product	64 7.2 [5.6–9.2]	72 8.0 [6.3–10.0]	110 8.4 [6.9–10.1]
Cigarettes + *IQOS*	40 4.5 [3.2–6.1]	62 6.9 [5.3–8.8]	62 4.8 [3.6–6.1]
Cigarettes + e-cigarettes	13 1.5 [0.7–2.5]	15 1.7 [0.9–2.8]	11 0.8 [0.4–1.6]
*IQOS* + e-cigarettes	5.0 0.6 [0.1–1.4]	4 0.4 [0.1–1.2]	15 1.2 [0.6–1.9]
*IQOS* + other product	4 0.5 [0.1–1.2]	2 0.2 [0.0–0.9]	11 0.8 [0.4–1.6]
E–cigarettes + other product	1 0.1 [0.0–0.7]	-	8 0.6 [0.2–1.3]
Two other products	–	–	1 0.1 [0.0–0.5]
Poly use	19 2.1 [1.2–3.4]	55 6.1 [4.6–7.9]	131 10.0 [8.4–11.9]
Cigarettes + *IQOS* + e-cigarettes	10 1.1 [0.5–2.1]	16 1.8 [1.0–2.9]	36 2.8 [1.9–3.9]
Cigarettes + *IQOS* + other product(s)	4 0.5 [0.1–1.2]	7 0.8 [0.3–1.6]	18 1.4 [0.8–2.2]
Cigarettes + e-cigarettes + other product(s)	3 0.3 [0.0–1.0]	18 2.0 [1.1–3.2]	45 3.5 [2.5– 4.6]
Cigarettes + other products	2 0.2 [0.0–0.9]	1 0.1 [0.0–0.7]	8 0.6 [0.2–1.3]
Cigarettes + *IQOS* + e-cigarettes + other product(s)	–	9 1.0 [0.4–1.9]	18 1.4 [0.8–2.2]
*IQOS* + e-cigarettes + other product(s)	–	4 0.4 [0.1–1.2]	5 0.4 [0.1–0.9]
*IQOS* + other products	–	–	1 0.1 [0.0–0.5]
E-cigarettes + other products	–	–	–
Three or more other products	–	–	–
Undefined	11 1.2 [0.6–2.3]	15 1.7 [0.9–2.8]	37 2.8 [2.0–3.9]

Abbreviations: CI, confidence interval; JGAP, representative sample of the Japanese general adult population. *Cigarettes include hand-rolled cigarettes

Across Y1-Y3 ( Table 4), the greatest proportion, although declining, of participants who reported TNP use were exclusive cigarette smokers (Y1-Y3: 79.5%, 68.1%, and 63.0%), while conversely, the proportion of exclusive *IQOS* users increased (Y1-Y3: 2.5%, 4.8%, and 5.3%), and that of exclusive e-cigarette users remained low (Y1-Y3: 0.3%, 1.1%, and 0.5%).

***Frequency and Intensity of TNP use***

Among the participants in the JGAP samples in Y1-Y3 who were currently using cigarettes (Y1-Y3: n=852; n=825; and n=1,150), the average number of cigarettes smoked per day (over the last 30 days) appeared to be stable (Y1-Y3: 16.0, 15.7, and 15.5) ( Table 5).

**Table 5.** Frequency and intensity of cigarette consumption among current cigarette smokers in the JGAP samples and of *HEETS*™ */HeatSticks*™ consumption among current *IQOS* users in the JA *IQOS* samples across the three study years.

	Mean [95% CI]		
	Year 1 *	Year 2	Year 3
Current cigarette smokers — JGAP	(n=852)	(n=825)	(n=1,150)
Cigarettes smoked per day			
Number of days of cigarette smoking in the last 30 days	29.4 [29.1–29.7]	29.2 [28.9–29.5]	29.3 [29.0–29.5]
Average number of cigarettes smoked per day (based on smoking days only)	16.2 [15.5–16.8]	15.9 [15.3–16.6]	15.8 [15.3–16.3]
Average number of cigarettes smoked per day in terms of the last 30-day period	16.0 [15.3–16.6]	15.7 [15.1–16.4]	15.5 [14.9–16.0]
Current *IQOS*™ users — JA *IQOS*	(n=2,000)	(n=2,044)	(n=2,013)
*HEETS*™/ *HeatSticks*™ used per day			
Number of days of *IQOS*™ use in the last 30 days	29.1 [28.9–29.3]	28.9 [28.7–29.1]	28.8 [28.5–29.0]
Average number of *HEETS/HeatSticks* used per day (based on usage days only)	16.2 [15.8–16.6]	16.5 [16.1–16.9]	15.9 [15.5–16.3]
Average number of *HEETS/HeatSticks* used per day in terms of the last 30-day period	15.9 [15.5–16.3]	16.1 [15.7–16.5]	15.5 [15.1–15.9]

Abbreviations: CI, confidence interval; JA *IQOS*, sample of adult Japanese *IQOS*™ users from PMI’s *IQOS* owner database in Japan; JGAP, representative sample of the Japanese general adult population; PMI, Philip Morris International * Year 1 (2016/2017), Year 2 (2017/2018), Year 3 (2018/2019)

***TNP initiation/relapse/reinitiation***

Among the participants who were never TNP users 12 months prior to the survey (Y1-Y3: n=3,066; n=3,109; and n=4,685), TNP use initiation with cigarettes in the preceding 12 months was considerably higher (Y1-Y3: 0.2%, 0.3%, and 0.2%) than initiation with *IQOS*™ (Y1-Y3: 0.03%, 0.1%, and 0.1%) ( Table 6).

**Table 6.** TNP initiation in JGAP samples among never TNP users.

	Never TNP Users Number (n) and percentage (% [95% CI])		
	Year 1 * (n=3,066)	Year 2 (n=3,109)	Year 3 (n=4,685)
Initiation with			
Cigarettes **	7 0.2 [0.0–0.5]	9 0.3 [0.1–0.6]	10 0.2 [0.1–0.4]
*IQOS*™ with *HEETS*™ */HeatSticks*™	1 0.03 [0.0–0.2]	4 0.1 [0.0–0.4]	5 0.1 [0.0–0.3]

Abbreviations: CI, confidence interval, JA *IQOS*, sample of adult Japanese *IQOS*™ users from PMI’s *IQOS* owner database in Japan; JGAP, representative sample of the Japanese general adult population; NA, not applicable; PMI, Philip Morris International; TNP, tobacco or nicotine-containing product. *Year 1 (2016/2017), Year 2 (2017/2018), and Year 3 (2018/2019) ** Cigarettes include hand-rolled cigarettes **Note:** Initiation with e-cigarettes was not measured as part of the study.

Among current TNP users in Y1-Y3 (Y1-Y3: n=894; n=900; and n=1,304), in each year only one participant reinitiated TNP use with *IQOS*™ (Y1-Y3: 0.1%, 0.1%, and 0.07%). No relapse to *IQOS* use was reported in any of the three study years ( Table 7).

**Table 7.** Relapse and reinitiation of TNP use with *IQOS*™ among current TNP users in the JGAP samples.

	Current TNP users Number (n) and percentage (% [95% CI])		
	Year 1 * (n=894)	Year 2 (n=900)	Year 3 (n=1,304)
Relapse to *IQOS*™	0 0.0 [0.0–0.5]	0 0.0 [0.0–0.5]	0 0.0 [0.0–0.3]
Reinitiation with *IQOS*	1 0.1 [0.0–0.7]	1 0.1 [0.0–0.7]	1 0.07 [0.0–0.5]

Abbreviations: CI, confidence interval; JGAP, representative sample of the Japanese general adult population; TNP, tobacco or nicotine-containing product. *Year 1 (2016/2017), Year 2 (2017/2018), and Year 3 (2018/2019)

**TNP use in the JA *IQOS* samples**

***Patterns of TNP Use***

Across Y1-Y3, a decreasing majority of participants in the samples used *IQOS*™ exclusively (Y1-Y3: 63.4%, 52.3%, and 49.4%), while the proportion who used *IQOS* together with other smoke-free TNPs increased (Y1-Y3: 7.6%, 17.7%, and 27.0%) and the proportion who used *IQOS* together with combustible TNPs decreased (Y1-Y3: 28.4%, 25.4%, and 23.6%). Consequently, by Y3, a greater proportion of participants used *IQOS* together with other smoke-free TNPs than *IQOS* together with combustible TNPs ( Table 8).

**Table 8.** Distribution of TNP use patterns in the JA *IQOS* sample.

	Number (n) and percentage (% [95% CI])		
	Year 1 * (n=1,946)	Year 2 (n=1,972)	Year 3 (n=1,977)
*IQOS*™ only	1,234 63.4 [61.2–65.6]	1,032 52.3 [50.1–54.6]	976 49.4 [47.1–51.6]
*IQOS* + combustible TNP	552 28.4 [26.3–30.5]	501 25.4 [23.4–27.4]	467 23.6 [21.7–25.6]
*IQOS* + smoke-free TNP	148 7.6 [6.4–8.9]	350 17.7 [16.0–19.6]	534 27.0 [25.0–29.1]
Undefined	12 0.6 [0.3-1.1]	89 4.5 [3.6-5.6]	0

Abbreviations: CI, confidence interval; JA *IQOS*, sample of adult Japanese *IQOS* users from PMI’s *IQOS*™ owner database in Japan; JGAP, representative sample of the Japanese general adult population; PMI, Philip Morris International; TNP, tobacco or nicotine-containing product. *Year 1 (2016/2017), Year 2 (2017/2018), and Year 3 (2018/2019)

***Frequency and intensity of TNP use***

In each of Y1-Y3 ( Table 5), the average number of days of *IQOS*™ use in the last 30 days (Y1-Y3: 29.1, 28.9, and 28.8) and the average number of *HEETS*™ */HeatSticks*™ used on the days of *IQOS* use in the last 30 days (Y1-Y3: 16.2, 16.5, and 15.9) were relatively stable. Thus, the average daily *HEETS/HeatSticks* consumption (over the last 30 days) across Y1-Y3 was similarly stable (Y1-Y3: 15.9, 16.1, and 15.5).

***History of TNP use***

In each of Y1-Y3, the majority of the JA *IQOS* sample participants had a smoking history before starting *IQOS*™ use (Y1-Y3: 98.0%, 98.7%, and 99.3%), while only a few were never smokers (Y1-Y3: 2.0%, 1.3%, and 0.7%) before starting *IQOS* use.

## Discussion

The present study is the first to report data on the prevalence and patterns of TNP use in samples of the Japanese general adult population (JGAP) and samples of Japanese adult *IQOS* users (JA *IQOS*) from a large *IQOS* user database over the same three consecutive years (2016/2017, 2017/2018, and 2018/2019). The findings of this study are consistent with the trends observed by other surveys that have examined the prevalence and patterns of TNP use since the introduction of the HTP *IQOS*™ in Japan in 2014 ^27– 31^.

In the JGAP samples, the prevalence of overall TNP use was stable (~18%) across the study years. However, there was a trend towards a declining prevalence of cigarette smoking concurrent with an increase in smoke-free TNP and total HTP use, especially in the case of *IQOS*™ use (from 1.8% in 2016/2017 to 3.3% in 2018/2019).

Although the cross-sectional data of the present study does not allow to draw cause-effect conclusions, given the trends observed, it can be hypothesized that the introduction of smoke-free TNPs does not lead to an unintended increase in overall TNP use in the general adult population, but rather drives a shift in TNP use patterns from cigarettes to smoke-free TNPs. When considered alongside the low TNP use initiation rates with *IQOS*™, the present findings further imply that the introduction of smoke-free TNPs has not led to an unintended increase in TNP use among adult non-users. This assumption is partially supported by the findings of other studies ^32, 33^ and is consistent with the findings of Cummings *et al.* ^4^, who reported an accelerated reduction in cigarette sales in Japan concurrent with the introduction and increase in HTP sales. In agreement with Cummings *et al.* ^4^ and others ^30, 31^, the total HTP use prevalence in year 3 of the present study was over 5%. A previous study had reported a total HTP use prevalence of 11% ^34^. The prevalence of cigarette smoking observed in each year of the present study was in agreement with the prevalence data from the Japan National Health and Nutrition Survey for 2017 (18.8%) ^27^, 2018 (18.9%) ^28^, and 2019 (17.7%) ^29^. Additionally, the present cigarette and overall TNP use data were well in line with those from other contemporaneous surveys ^30, 31, 35, 36^. Tabuchi *et al.* ^32^ reported an *IQOS*™ use prevalence of 3.6% in 2017, which is higher than that observed in the present study (1.8%) for the same year. For 2018, Sutanto *et al.* ^37^ reported an any-brand HTP use prevalence (i.e., *IQOS*™, glo, and Ploom/Ploom Tech) of 2.7%, which is less than that observed in our study (3.2%) for *IQOS* use alone. These discrepancies are likely due to methodological differences (i.e., cross-sectional vs. longitudinal design or in-person vs. online interview) as described previously ^23^.

The above mentioned rather large difference in total HTP use prevalence between our study (5%) and the study of Hori *et al.* (11%) ^34^, respectively, may have resulted from multiple conceptual and methodological differences including i.a., (i) cross-sectional vs. longitudinal design, (ii) in-person vs. online interview (iii) age ≥20 years vs. age range 15-69 years, (iv) no weighting methods vs. inverse probability weighting, (v) no adjustments vs. adjustment of the data, (vi) definition of current use [at the time of the survey vs. past 30 days], and (vii) lifetime use criterion [100 tobacco sticks] vs. no lifetime use criterion to qualify as a HTP user. Of all these aspects, however, we believe that the difference in the age groups included in both studies (i.e., age ≥20 year in our study vs. 15-69 years in the Hori *et al.* study ^34^) with very low HTP use prevalence in 60+ age groups, had the largest impact on the difference in the total HTP prevalence observed between our study and the study of Hori *et al.* ^34^

In the JGAP samples, the age and sex distribution observed among current tobacco product users in each of the study year as well as the current HTP use patterns are similar to the corresponding data of the Japan National Health and Nutrition Survey for the same years ^27– 29^. Similarly, in agreement with the Japanese national survey ^27– 29^, the results presented here indicate that the majority of HTP users are using HTP exclusively. The average number of *HEETS*™ */HeatSticks*™ used per day (Y1-Y3: 15.9, 16.1, and 15.5, respectively) in the JA *IQOS* samples was relatively stable across the study years and comparable with the 14.3 *HEETS/HeatSticks* used per day reported for Japan by Jones *et al.* ^31^ in 2019. Moreover, the *HEETS/HeatSticks* consumption per day in the JA *IQOS* samples was very close to the average number of cigarettes consumed per day (Y1-Y3: 16.0, 15.7, and 15.5, respectively) among cigarette smokers in the JGAP samples, suggesting that *IQOS* users are not increasing their daily consumption upon switching from cigarettes to *IQOS*™.

TNP initiation, relapse, and reinitiation rates observed with *IQOS*™ in the present study were in all three years and in both samples relatively low, suggesting that *IQOS* uptake was limited to existing smokers who had switched to *IQOS*. Similarly, Sutanto *et al.* ^37^ concluded that “ *virtually all HTP users were current cigarette smokers (67.8%) or former smokers (25.0%); and that only 1.0% of HTP users were never smokers*.” Jones *et al.* ^31^ observed that HTP uptake in 2019 occurred “ *almost exclusively among current tobacco users in Japan, with negligible uptake among never tobacco users*.” In both samples in the present study, nearly all current *IQOS* users had started TNP use with cigarette smoking. These findings suggest that *IQOS* uptake is occurring among current adult smokers, which is in alignment with both the principles of harm reduction and the United States Food and Drug Administration’s (FDA) conclusion that *IQOS* has “ *potential benefit to population health*” ^20^.

Our study is based on Japanese adult participants only, but with regard to tobacco harm reduction, also unintended uptake of *IQOS*™/HTPs among youth and young adults may be of concern. In the literature, tobacco initiation with *IQOS*/HTPs and prevalence of *IQOS*/HTP use among youth has been reported to be low in Japan. Based on data from the representative longitudinal internet-based JASTIS study in Japan, the 2015, 2016, and 2017 prevalence of ever use of *IQOS* with *HEETS*™/ *HeatSticks*™ among youth (15–19 years) in the past 30 days was estimated to be 0.6%, 2.3%, and 2.0%, respectively ^32^. Similarly, in a nationwide survey in Japan among middle-school and high-school students, respectively, the estimated 2017/2018 prevalence for ever-HTP use (even one puff) in lifetime (1.1% and 2.2%), ever HTP use in the past 30 days (0.5% and 0.9%), and daily HTP use (0.1% and 0.1%) was very low ^38^.

Lastly, regarding the decline in response rates observed for the JA *IQOS* samples across the three study years, this decline can be explained by the fact that over time the *IQOS* users increasingly received email invitations to various PMI surveys which probably has generated a lower willingness to participate in our scientific surveys.

### Strengths and limitations

Major strengths of this study, among those previously described ^23^, include the annual repeated data collection using the same sampling framework and methods, face-to-face interviews, and nationally representative samples.

The limitations of the current study include all biases typically associated with self-reported measures, such as recall, social desirability, or response bias as well as sampling and selection bias. To overcome these limitations, particularly in the JA *IQOS* sample, the response rates were monitored and compared against preestablished quotas on the basis of age and sex.

The limitations of the current study include foremost the cross-sectional and observational design that does not allow for cause-effect inference or investigation of switching/transition behaviors over time. Moreover, the study may have suffered from all biases typically associated with self-reported measures, such as response bias including i.a., recall, social desirability, or order effect bias, as well as sampling and selection bias. To overcome possible sampling and selection bias, particularly in the JA *IQOS* samples, the response rates were monitored and compared against pre-established quotas on the basis of age and sex. Furthermore, the JA *IQOS* samples, which we used to obtain reliable estimates for investigating the patterns of *IQOS*™ use, were not representative of the *IQOS* users in the Japanese general adult population, but only of the *IQOS* users registered in the large database of PMI’s affiliate in Japan. Similarly, because *IQOS* was the first HTP and had the highest use prevalence in Japan, *IQOS* use behavior may not have represented the use behavior of other HTPs available in Japan. Finally, the present study neither addresses quitting behavior, e.g., investigation of whether *IQOS*™/HTP use prevents those TNP users who are willing to quit all TNPs to do so, nor does the study cover *IQOS*/HTP underage use, which are critical aspects for assessing the impact of *IQOS*/HTP use on tobacco harm reduction.

## Conclusions

While cigarette smoking remains the most prevalent way of consuming TNPs in Japan, a significant and growing number of adult Japanese smokers have switched to smoke-free alternatives such as *IQOS*™ *,* with the majority using these products exclusively. Additionally, there has been low initiation with *IQOS* among TNP never and former users. Taken together, the findings of the present study on the prevalence and patterns of *IQOS* use indicate that the trends in *IQOS* use behavior suggest that *IQOS* has the potential to switch adult smokers from cigarettes to smoke-free tobacco products, which presents a harm reduction opportunity, and that HTPs are effective tools for complementing current tobacco control efforts ^14^.

## Data Availability

INTERVALS: YEAR 1–3 DATA (SAS DATASETS, CC-BY 4.0), https://doi.org/10.26126/intervals.r35iml.1 ^39^ This project contains the following underlying data: SAS datafiles in the Clinical Data Interchange Standards Consortium (CDISC) Analysis Data Model (ADaM) structure ( www.cdisc.org/standards) for each of the study years 1–3. The ADSL (adsl_y1_jp.sas7bdat, adsl_y2_jp.sas7bdat, and adsl_y3_jp.sas7bdat,) datasets are the Subject Level Analysis Datasets and contain the main information on participants identifier, demographics, and tobacco and/or nicotine product use groups and patterns to facilitate analysis and interpretation of analysis. The ADQS (adqs_y1_jp.sas7bdat, adqs_y2_jp.sas7bdat, and adqs_y3_jp.sas7bdat) datasets are the Questionnaire Analysis Datasets and contain specific information on the study survey, i.e., all questions and items answered by participants in the survey. The ADEX (adex_y1_jp.sas7bdat, adex_y2_jp.sas7bdat, and adex_y3_jp.sas7bdat) datasets are the Exposure Analysis Datasets and contain specific information on the TNP use exposure, i.e., all questions and items answered by participants in the survey related to their product use. The ADAM Metadata Files (ADaM_PMX01JP_AnY1_Metadata, ADaM_PMX01JP_AnY2_Metadata, and ADaM_PMX01JP_AnY3_Metadata) contain the datasets and variable labels and definitions, code lists to decode the variables names, terms and values, and the methods and computational algorithms to derive the analytical datasets. Study Year 1 Data and Metadata: https://doi.org/10.26126/intervals.8ybcxu Study Year 2 Data and Metadata: https://doi.org/10.26126/intervals.hxaf2v Study Year 3 Data and Metadata: https://doi.org/10.26126/intervals.6jbifs INTERVALS: Tobacco Use Prevalence Questionnaire. https://doi.org/10.26126/intervals.rxhx4a.1 ^39^ This project contains the following extended data: Tobacco Use Prevalence questionnaire (Tobacco Use Prevalence Questionnaire_engl_jap.pdf) is the questionnaire administered in the general population and *IQOS* user surveys (English and Japanese version). Data are available under the terms of the Creative Commons Attribution 4.0 International license (CC-BY 4.0). This study followed the STROBE reporting guideline. INTERVALS: STROBE checklist and flow chart for “Trends in Prevalence and Patterns of Use of a Heated Tobacco Product ( *IQOS*™) in Japan: A 3-Year Repeated Cross-Sectional Study”, are available: https://doi.org/10.26126/intervals.dluspw
