# Supplementary material for: Trends in prevalence and patterns of use of a heated tobacco product ( IQOS ™) in Japan: A three-year repeated cross-sectional study
Source: F1000Res. 2022 Nov 21;11:720. Originally published 2022 Jun 30. [Version 2] doi: 10.12688/f1000research.122491.2 (PMC12716265; doi:10.12688/f1000research.122491.2)
Supplement: Peer review history

# Reviewer report: Reviewer response for version 2

**DOI:** 10.5256/f1000research.140767.r381666

**Published:** 2025-05-19

**Authors:** Delfin Lovelina Francis

**Affiliations:** 1 Saveetha University, Chennai, Tamil Nadu, India

General Overview:

 The manuscript addresses an important and timely topic on trends in the prevalence and patterns of use of heated tobacco products, mainly IQOS, in Japan.  Although the methodology is solid, the study was repeated cross-sectional over three years. The findings give good insight in the changing picture of tobacco consumption. The literature review is well prepared, the data presented are informative. There are several areas that require clarification and improvement to reach stronger and clearer conclusions.

 Strengths:

 1. As the use of heated tobacco products, especially IQOS, continues to grow around the world, the study adds important evidence on the penetration of such products in Japan. Moreover, the selection of a three-year study period is a strength because it enables an assessment of changes over time.

 2. The design chosen for the study – repeated cross-sectional is appropriate for observing trends over periods of time. Also, the statistical methods applied seem to be adequate. The manuscript explains fair methodology and demographical profile of the sample which improves the transparency of the study.

 3. The results of the study suggest clearly public health policy implications, including the need for continued surveillance and regulation of new tobacco products. The manuscript points out that IQOS has been used increasingly and that surveillance and regulation remains a policy consideration.

**Areas for Improvement:**

 1. The results section could benefit from more detailed breakdowns of the data, particularly in terms of sub-group analyses. I believe it would be useful to explore variations in use of IQOS by various demographic variables (age, sex, smoking history) although the population studies include these in the main conclusions but would offer more detailed insight into the patterns.

 2.The use of terms like ever use and current use should be clearly indicated in the methods section, to avoid confusion, most probably where these categories are used in the results as synonymous.

 3.The manuscript could be better developed in terms of potential limitations of the study design, such as the use of self-reported data; although the authors acknowledge that there are potential biases, the need to be greater discussion of the potential impact of the limitation on the results, particularly in the case of HTP use when social desirability bias may be present.

 4.The authors should consider whether the introduction of marketing strategies (e. g. targeted ads, promotions) might affect the observed trends in use because such external factors may have a significant effect on uptake and use patterns.

 5.The manuscript briefly mentions the prevalence of IQOS usage, but more should be said about the reasons for this. Are there particular socio-cultural factors that might explain the high prevalence of IQOS in Japan compared to other countries? These aspects might make the manuscript relevant and provide more detailed analysis.

 6.It would be helpful to compare the trends examined in Japan with trends observed elsewhere, and particularly with regard to the global distribution of HTPs since this would contextualize the results and provide further insight into the factors that may influence IQOS use.

 7.Further comparison with other forms of tobacco use (e. g., e-cigarettes) may also be necessary to shed more light on the relative appeal of IQOS and its replacement or complementarity with other forms of tobacco.

 This study provides important insights on the trends in the use of IQOS (including implications for tobacco control policy) in Japan. Although, it is a good publication it would benefit from a more detailed discussion of the results, limitations, and potential policy recommendations. A more detailed understanding of the factors influencing HTP use and a comparative study with other countries would thus add value to the manuscript.

Is the work clearly and accurately presented and does it cite the current literature?

Yes

If applicable, is the statistical analysis and its interpretation appropriate?

I cannot comment. A qualified statistician is required.

Are all the source data underlying the results available to ensure full reproducibility?

Yes

Is the study design appropriate and is the work technically sound?

Partly

Are the conclusions drawn adequately supported by the results?

Yes

Are sufficient details of methods and analysis provided to allow replication by others?

Yes

Reviewer Expertise:

Tobacco control, cancer epidemiology

I confirm that I have read this submission and believe that I have an appropriate level of expertise to confirm that it is of an acceptable scientific standard, however I have significant reservations, as outlined above.

# Reviewer report: Reviewer response for version 2

**DOI:** 10.5256/f1000research.140767.r156232

**Published:** 2023-05-11

**Authors:** Mohamadi Sarkar, Brendan Noggle

**Affiliations:** 1 Center for Research and Technology, Altria Client Services LLC, Richmond, VA, USA; 2 Center for Research and Technology, Altria Client Services LLC, Richmond, VA, USA

The authors have adequately addressed the comments and I approve this manuscript.

Is the work clearly and accurately presented and does it cite the current literature?

Yes

If applicable, is the statistical analysis and its interpretation appropriate?

Partly

Are all the source data underlying the results available to ensure full reproducibility?

Yes

Is the study design appropriate and is the work technically sound?

Yes

Are the conclusions drawn adequately supported by the results?

Partly

Are sufficient details of methods and analysis provided to allow replication by others?

Yes

Reviewer Expertise:

Tobacco regulatory science.

We confirm that we have read this submission and believe that we have an appropriate level of expertise to confirm that it is of an acceptable scientific standard.

# Reviewer report: Reviewer response for version 1

**DOI:** 10.5256/f1000research.134482.r144380

**Published:** 2022-09-21

**Authors:** David T. Levy

**Affiliations:** 1 Lombardi Comprehensive Cancer Center, Georgetown University, Washington, DC, USA

This study provides interesting results on trends in IQOS use as related to smoking and vaping. The methodology and results are well presented. With regards to data underlying the results, I don’t recall that the authors provided for data transferred to others. While desired, there may be issues of confidentiality. Using cross-sectional data, their results show increasing use of IQOS with and without e-cigarettes and a decline in smoking. Their results, however, in the Discussion section, I had problems with the failure to adequately discuss the limitations of the study and the author’s generalization of the results.

 The limitations of the study merit further attention in the Discussion section.  The authors fail to recognize the limited nature of their sample. In particular, the sample is limited to IQOS users and the sample is based on IQOS users who chose to register on the PMI website, thus presenting the potential problem of selection bias. Indeed, IQOS was the first and has a dominant share in Japan and may not represent other HTP use and its relationship to smoking. These limitations should be clearly recognized and discussed.  It wasn’t clear to me whether there was a switching between IQOS and other HTPs.  The use of cross-sectional rather than longitudinal data also merits discussion.

 In addition, the authors attempt to generalize the results for IQOS and e-cigarette use in their sample to the rest of the Japanese population by comparing to NHNS and other samples. As currently presented, I did not feel that the conclusions, ie. Upward exclusive use of IQOS as cigarette use decline, are well supported. The authors do compare some trends, but the comparisons are not rigorous. It would be useful to compare relative changes over time in their sample to relative changes other more representative samples (e.g., NHNS, JASTIS), and provide direct comparisons by age and gender in a table. The authors may then be able to compare whether the trends related to IQOS and cigarette use are the same or different. Given the potential for selection bias, which should be made explicit, these comparisons are important.

Is the work clearly and accurately presented and does it cite the current literature?

Partly

If applicable, is the statistical analysis and its interpretation appropriate?

Partly

Are all the source data underlying the results available to ensure full reproducibility?

No

Is the study design appropriate and is the work technically sound?

Partly

Are the conclusions drawn adequately supported by the results?

No

Are sufficient details of methods and analysis provided to allow replication by others?

Yes

Reviewer Expertise:

Modelling and empirical analyses

I confirm that I have read this submission and believe that I have an appropriate level of expertise to confirm that it is of an acceptable scientific standard, however I have significant reservations, as outlined above.

# Response

**Published:** 2022-11-04

**Authors:** Karina Fischer

**Affiliations:** Philip Morris, Switzerland

**Author Responses to Comments of Reviewers**

 We thank Reviewer 1 and Reviewers 2 for their critical and comprehensive review as well as their constructive suggestions to improve the manuscript. We addressed all comments by point-to-point responses and referred to the related updates in the manuscript by page and line numbers ( Note: *page and line numbers reflect the tracked changes version 2 of the manuscript, not the edited version that reviewers will receive. Reviewers may have to additionally request the tracked changes version*).

**Reviewer 1:** *David T. Levy Lombardi Comprehensive Cancer Center, Georgetown University, Washington, DC, USA.*

**Reviewer 1 comments**

**Reviewer 1 Comment 1.** This study provides interesting results on trends in IQOS use as related to smoking and vaping. The methodology and results are well presented. With regards to data underlying the results, I don’t recall that the authors provided for data transferred to others. While desired, there may be issues of confidentiality.

***Authors’ Response 1*** **.** We thank Reviewer 1 for this comment. All data and extended data underlying the results and conclusions of this study as well as the questionnaire and reporting guideline related information (Strobe Checklist) have already been made publicly available on the INTERVALS platform as part of the manuscript submission to F1000Reserach (see **Data Availability section [pages 25-26] of the manuscript**). Therefore, all data and information underlying the manuscript, are publicly available on the INTERVALS platform and have a Digital Object Identifier (DOI) that directs to the related data/information on INTERVALS.

**Reviewer 1 Comment 2.** Using cross-sectional data, their results show increasing use of IQOS with and without e-cigarettes and a decline in smoking. Their results, however, in the Discussion section, I had problems with the failure to adequately discuss the limitations of the study and the author’s generalization of the results.

***Authors’ Response 2.*** We agree with Reviewer 1 to highlight the limitations of the study in more detail. We amended the discussion (page 22, lines 305-308) and limitation (page 24, lines 382-384) section with regard to the limitations of the cross-sectional design, as well as the limitation section in view of the limited generalizability of the results from the *IQOS *user (JA *IQOS*) samples (page 24, lines 389-392).

**Reviewer 1 Comment 3.** The limitations of the study merit further attention in the Discussion section. The authors fail to recognize the limited nature of their sample. In particular, the sample is limited to IQOS users and the sample is based on IQOS users who chose to register on the PMI website, thus presenting the potential problem of selection bias. Indeed, IQOS was the first and has a dominant share in Japan and may not represent other HTP use and its relationship to smoking. These limitations should be clearly recognized and discussed. It wasn’t clear to me whether there was a switching between IQOS and other HTPs. The use of cross-sectional rather than longitudinal data also merits discussion.

***Authors’ Response 3.*** We would like to clarify that In each of the three study years, the study included both (i) ***representative*** samples of the ***Japanese General Adult Population*** ( **JGAP** samples based on **three-stage stratified proportional random sampling** covering the whole of Japan) as well as (ii) “ **convenience”** samples of Japanese adult *IQOS* users registered in the *IQOS* owner database of Philip Morris International’s affiliate in Japan ( **JA *IQOS*** samples ***randomly selected*** from about 350,000 *IQOS* users in 2017 and 6 million *IQOS* users in 2019 ***registered in the Japanese adult IQOS owner database***). As stated at the end of the introduction, we used the *IQOS* user samples (JA *IQOS* samples) alongside the representative JGAP samples because in the first and second year of the study *IQOS* was relatively new on the Japanese tobacco market, and thus, the *IQOS *use prevalence in the general population (representative JGAP samples) was expected to be low. Therefore, to obtain reliable estimates of the use patterns of *IQOS* (i.e., exclusive *IQOS* use, *IQOS* use with combustible tobacco products, and *IQOS* use with non-combustible tobacco products, the differentiation of which is important regarding the impact on tobacco harm reduction), we conducted additional surveys among *IQOS* users.

 Because we are aware of the limitations of the JA *IQOS* samples, most of the results presented in our study are based on the representative JGAP samples (Tables 1-7 and the related text in the results section), whereas only the results presented in Table 1 (demographics), Table 5 (frequency and intensity of use) and Table 8 ( *IQOS* use patterns) are also presented for the *IQOS* user samples.

 Both (i) the representative samples of the Japanese General Adult Population (JGAP samples) as well as (ii) the samples of Japanese adult *IQOS* users registered in Japanese the *IQOS *owner database (JA *IQOS* samples) were random samples. Therefore, we believe that the likelihood of selection bias was rather low.

 Because the present study provides cross-sectional but not longitudinal data of the same participants over time, investigating switching behaviors was not intended for this manuscript.

 As already stated in our response to comment 2, we amended the discussion (page 22, lines 305-308) and limitation section (page 24, lines 382-384) with regard to the limitations of the cross-sectional design, as well as the limitation section in view of the limited generalizability of the results from the *IQOS* user (JA *IQOS*) samples (page 24, lines 389-392). Moreover, in the limitation section we added the fact even though *IQOS*™ was the first HTP and had the highest use prevalence in Japan, *IQOS* use behavior may not represent the use behavior of other HTPs available in Japan (page 24, lines 392-394).

**Reviewer 1 Comment 4.** In addition, the authors attempt to generalize the results for IQOS and e-cigarette use in their sample to the rest of the Japanese population by comparing to NHNS and other samples. As currently presented, I did not feel that the conclusions, i.e. Upward exclusive use of IQOS as cigarette use decline, are well supported. The authors do compare some trends, but the comparisons are not rigorous. It would be useful to compare relative changes over time in their sample to relative changes other more representative samples (e.g., NHNS, JASTIS), and provide direct comparisons by age and gender in a table. The authors may then be able to compare whether the trends related to IQOS and cigarette use are the same or different. Given the potential for selection bias, which should be made explicit, these comparisons are important.

***Authors’ Response 4.*** We thank Reviewer 1 for raising this point. Regarding the prevalence data of overall tobacco product as well as *IQOS* and other individual tobacco product use, in the discussion, we compared the study results from the representative JGAP samples with the corresponding data from one of the most comprehensive surveys measuring tobacco use, the representative Japan National Health and Nutrition Survey (Japan NHNS) from the Japanese Ministry of Health, Labour and Welfare.

 While we indeed present data on exclusive *IQOS* use, we did not state that with increasing exclusive *IQOS* use the use of cigarettes declined as Reviewer 1 remarked. Rather, in the manuscript (page 22, lines 302-304) we stated “there was a trend towards a declining prevalence of cigarette smoking concurrent with an increase in smoke-free TNP and total HTP use, especially in case of *IQOS* use. So, with regard to the decline in overall cigarette smoking prevalence, we are not referring to exclusive *IQOS* use but to overall smoke-free TNP, total HTP, and *IQOS* use.

 Regarding the point that our survey may have suffered from selection bias (see also our response on selection bias to Reviewer 1’s comment 3), we mentioned this aspect in the limitation section (page 24, line 386-389). However, because both (i) the ***representative*** samples of the ***Japanese General Adult Population*** ( **JGAP** samples, ***three-stage stratified proportional sampling***) as well as (ii) the samples of Japanese adult *IQOS* users registered in Japanese adult *IQOS* owner database ( **JA *IQOS*** samples ***randomly selected*** from about 350,000 *IQOS* users in 2017 and 6 million *IQOS* users in 2019 registered in the Japanese adult *IQOS* owner database) were random samples from the respective reference populations, we believe that the likelihood of selection bias was rather low.

 As opposed to our *cross-sectional* study that includes adult participants ≥20 years and presents unadjusted data, the Japan Society and New Tobacco Internet Survey (JASTIS) is a *longitudinal* (cohort) study following both youth and adults aged 15-73 years over time and presents adjusted data. Therefore, given the different study design and age groups included, we cannot directly compare our results from the cross-sectional JGAP and JA *IQOS* samples with the longitudinal JASTIS study as Reviewer 1 suggested ( *see also Authors’ Response to Reviewers 2 Minor Comment 2*). However, we compared our prevalence data from the representative JGAP samples with the sales data of cigarettes and heated tobacco product (HTP) use ( **Chart 1 below) **as well as with the prevalence data from the representative Japan National Health and Nutrition Survey (Japan NHNS) conducted yearly by the Japan Ministry of Health, Labour and Welfare ( **Chart 2 below**) over time.

 In 2021, HTP units represented almost 1/3 of the total cigarette and HTP market. The increase in HTP sales was accompanied by a decline in cigarette sales ( **Chart 1 below)**.

**Chart 1** Cummings K. F. et al.(2020) What Is Accounting for the Rapid Decline in Cigarette Sales in Japan? Int J Environ Res Public Health; 17(10): 3570. Doi: 10.3390/ijerph17103570. The sales data for 2020 and 2021 was added to the data published for 2011-2019 by Cummings et al.

 The prevalence data from the Japan NHNS ( **Chart 2 below**) suggests that the decline in cigarette sales shown in **Chart 1** was accompanied by a decrease in smoking prevalence. Until 2017, the Japan NHNS measured only the prevalence of smoking. However, because between 2015 and 2017 HTP sales had already rapidly increased as shown in **Chart 1**, it can be assumed that between 2015 and 2017 also the HTP use prevalence increased.

 In 2018 the Japan NHNS was updated to capture the prevalence of both cigarettes and HTPs separately. In 2019, 12% of the adult population were smoking only combustible cigarettes, shown in dark grey, 3.4% were using only HTPs, shown in light blue, 1.1% were using both cigarettes and HTPs, and 0.2% used other or undefined tobacco products ( **Chart 2 below**). Because the Japan NHNS was discontinued in 2020 and 2021 due to the Covid pandemic, there is unfortunately no data for more recent trends.

**Chart 2. **Prevalence data on cigarette and HTP use from the Japan National Health and Nutrition Survey (Japan NHNS) conducted by the Japan Ministry of Health, Labour and Welfare

 The data presented in Table 4 of our manuscript for cigarette and *IQOS* use prevalence in the representative GAP samples from 2016-2019 ( **shown as** **Chart 3 below**; the chart includes also the most recent 2020/2021 GAP data) are similar to the 2016 -2019 data from the Japan NHNS, which shows a decline in cigarette smoking prevalence accompanied with a marked uptake of HTP use as of 2018 when both cigarettes and HTPs were separately measured in the Japan NHNS.

**Chart 3.** Data presented in Table 4 of the manuscript for cigarette and IQOS use prevalence in the representative GAP samples from 2016 -2019 ( **on the left side of the green dashed line**) as well as most recent 2020/2021 prevalence data for cigarette and IQOS use ( **on the right side of the green dashed line**) that are not shown in the manuscript.

 Given all the information presented above, we believe that our observation of a decline in cigarette smoking prevalence accompanied with an increase in *IQOS/*HTP use prevalence over the three study years in Japan is supported by independent data.

# Reviewer report: Reviewer response for version 1

**DOI:** 10.5256/f1000research.134482.r142791

**Published:** 2022-07-28

**Authors:** Mohamadi Sarkar, Brendan Noggle

**Affiliations:** 1 Center for Research and Technology, Altria Client Services LLC, Richmond, VA, USA; 2 Center for Research and Technology, Altria Client Services LLC, Richmond, VA, USA

***17/10/22: The reviewers' COI statement, "The reviewers are employees of Altria Client Services LLC.", was updated to include more detail on the potential link between the funders of this research and the reviewers, which was not fully declared at the time of publishing of this report.***

 We read the manuscript submitted by Fischer *et al *in F1000Research with great interest. The authors report results from cross-sectional surveys conducted over a period of three in representative samples of the Japanese general adult population and samples of Japanese adult IQOS users registered in the IQOS owner database. The authors conclude that the trend towards declining prevalence of cigarette smoking concurrent with an increase in smoke-free TNP and total HTP use, especially in the case of IQOS ^TM^ use are in line with the principles of tobacco harm reduction and that HTPs are effective tools for complementing current tobacco control measures. We have the following major and minor comments as listed below. We recommend that the authors address each comment and accordingly modify the manuscript.

*Major comments*

1. The authors report results from the general population survey among Japanese adults, which given the representativeness is appropriate to compare over time.  However, the IQOS user survey was based on a convenience sample, and comparing trends over time is less robust. Therefore, comparing the results across the two surveys is not ideal.  The authors should discuss this as a limitation of their approach.  Instead of a direct comparison of the survey results between the general population and IQOS sample the authors should have considered characterizing IQOS sample tobacco use behaviors and benchmark that to the current adult tobacco users from the general population survey.
2. We were confused by the individual TNP use prevalence values among the general population reported in Table 3. The overall prevalence for cigarettes ranges from 17.6, 17.3, and 16 % each year. If add the prevalence values for IQOS and e-cigarettes, results in total TNP prevalence of 20.1, 22.1, and 21.3% for each year.  These values differ from the overall TNP prevalence of 18.5, 18.9, and 18.2 reported for each year in Table 2. Perhaps the authors can offer an explanation for this discrepancy
3. While we agree that this evidence supports or does not contradict that switching to a smoke-free product like IQOS supports harm reduction, we suggest that the authors should be careful in drawing broader conclusions like “IQOS use behavior trends are in line with the principles of tobacco harm reduction”. Tobacco harm reduction cannot be viewed only in the narrow perspective of switching.  An important consideration of unintended consequence is not only initiation among adults but also among youth. Additionally, as pointed in our comment above, the total TNP prevalence as calculated by us does not indicate a reduction in total tobacco product use, on the contrary, appears to increase over time.  The authors should provide some context regarding these observations. Therefore, the authors should consider modifying the conclusions as “The use behavior trends suggest that IQOS has the potential to switch adult smokers from cigarettes to smoke-free products, which presents a harm reduction opportunity.”  
4. We caution the authors to avoid making causality inferences based on association.  The authors should consider revising the conclusion - “The introduction of smoke-free TNPs does not lead to an unintended increase in overall TNP use in the general population, but rather drives a shift in TNP use patterns from cigarettes to smoke-free TNPs.” to reflect association rather than causation.
5. The survey lacks an appropriate characterization of quitting behavior among IQOS users.  The survey does not present evidence regarding the likely interception of smokers who may have otherwise quit using TNP. If the survey did not assess this domain, the authors should cite this as a limitation.  This further warrants a revision of the conclusions from not being so definitive but reflect the limitations of the survey results.  
6. The authors should include an explanation of the drop in response rates for JA *IQOS *samples from 19.4% in Y1 to 4.7% and 2.0% in Y2 and Y3. Given that the IQOS users changed significantly, from 350,000 to 6 million, the IQOS population likely changed over the course of the 3 surveys. The authors should consider accounting for the change in IQOS population by adjusting either during recruitment or during the data analysis.
7. The authors only present descriptive analyses of survey findings, however, they may want to consider including some trend analysis or other statistical testing to determine whether the changes over time are significant.  Perhaps such an analysis may not yield robust findings from a three-year survey and authors may consider providing more statistical rigor in future reports.
8.  We understand that the focus of this manuscript was among adults age >20 years.  However, an important consideration is the impact of IQOS and other smoke-free products among youth, which is an important public health concern.  The authors should mention that this manuscript only addresses adult use behavior and either briefly describe data for youth use based on published literature or national surveys. Some indication of initiation among different age groups of youth and young adults would have provided useful insights in the overall harm reduction potential of IQOS and other smoke-free products.

*Minor comments*

1. The authors should consider including the supporting literature citations in the statement – “The existing standard TNP use questions are available in the literature.”.
2. The authors should provide a possible explanation of the discrepancy in the reported total HTP use prevalence of 11% by Hori *et al *(ref 34) which is almost twice of that reported in the current manuscript.  This discrepancy cannot be solely explained by methodological differences.
3. In this manuscript, the IQOS sample could have been better characterized.  Given that the authors had access to the IQOS user database, they could have followed transition behavior over time rather than indirectly comparing changes in average smoking prevalence against increase in IQOS user prevalence. Moreover, they may also report transitions between dual use to exclusive switching behavior. Perhaps in future reports, the authors can include more detailed analyses of transition behaviors.
4. We believe that the title for Table 6 should include JGAP not JPAG. Please correct the typo.

Is the work clearly and accurately presented and does it cite the current literature?

Yes

If applicable, is the statistical analysis and its interpretation appropriate?

Partly

Are all the source data underlying the results available to ensure full reproducibility?

Yes

Is the study design appropriate and is the work technically sound?

Yes

Are the conclusions drawn adequately supported by the results?

Partly

Are sufficient details of methods and analysis provided to allow replication by others?

Yes

Reviewer Expertise:

Tobacco regulatory science.

We confirm that we have read this submission and believe that we have an appropriate level of expertise to confirm that it is of an acceptable scientific standard, however we have significant reservations, as outlined above.

# Response

**Published:** 2022-11-04

**Authors:** Karina Fischer

**Affiliations:** Philip Morris, Switzerland

**Author Responses to Comments of Reviewers**

 We thank Reviewer 1 and Reviewers 2 for their critical and comprehensive review as well as their constructive suggestions to improve the manuscript. We addressed all comments by point-to-point responses and referred to the related updates in the manuscript by page and line numbers ( Note: *page and line numbers reflect the tracked changes version 2 of the manuscript, not the edited version that reviewers will receive. Reviewers may have to additionally request the tracked changes version*).

**Reviewers 2:**

*Mohamadi Sarkar, Center for Research and Technology, Altria Client Services LLC, Richmond, VA, USA*

*Brendan Noggle, Center for Research and Technology, Altria Client Services LLC, Richmond, VA, USA*

**Reviewers 2 comments**

 We read the manuscript submitted by Fischer et al in F1000Research with great interest. The authors report results from cross-sectional surveys conducted over a period of three years in representative samples of the Japanese general adult population and samples of Japanese adult IQOS users registered in the IQOS owner database. The authors conclude that the trend towards declining prevalence of cigarette smoking concurrent with an increase in smoke-free TNP and total HTP use, especially in the case of *IQOS*™ use are in line with the principles of tobacco harm reduction and that HTPs are effective tools for complementing current tobacco control measures. We have the following major and minor comments as listed below. We recommend that the authors address each comment and accordingly modify the manuscript.

**Major comments**

**Reviewers 2** **Major Comment 1.** The authors report results from the general population survey among Japanese adults, which given the representativeness is appropriate to compare over time. However, the IQOS user survey was based on a convenience sample and comparing trends over time is less robust. Therefore, comparing the results across the two surveys is not ideal. The authors should discuss this as a limitation of their approach. Instead of a direct comparison of the survey results between the general population and IQOS sample the authors should have considered characterizing IQOS sample tobacco use behaviors and benchmark that to the current adult tobacco users from the general population survey.

***Authors’ Response Major Comment 1.*** As stated at the end of the introduction, we used the “convenience” JA *IQOS* samples (random samples from about 350,000 *IQOS* users in 2017 and 6 million *IQOS* users in 2019 registered in the adult *IQOS* owner database of PMI’s affiliate in Japan) alongside the representative JGAP samples because in the first and second year *IQOS* was relatively new on the Japanese tobacco market, and thus, the *IQOS *use prevalence in the representative JGAP samples was expected to be low. Therefore, to obtain reliable estimates of the use patterns of *IQOS* (e.g., exclusive *IQOS* use, *IQOS* use with combustible tobacco products, and *IQOS* use with non-combustible tobacco products, the differentiation of which is important regarding the impact on tobacco harm reduction), additional surveys among *IQOS* users were conducted.

 Although we presented the results of both samples in the results section, we only directly compared the study results on age and sex distribution, intensity of use, initiation, relapse, and reinitiation between the two samples because we believe it was important to show the study results of the two independent samples.

 Because we are well aware of the limitations of the *IQOS* user (JA *IQOS*) samples, most of the results presented are based on the representative JGAP samples (Tables 1 [demographics] and Tables 2-7) and the related text in the results section. Only the results presented in Table 1 [demographics], Table 5 [frequency and intensity of use] and Table 8 [ *IQOS* use patterns] are also presented for the *IQOS* user samples. We amended the discussion to highlight the limited generalizability of the *IQOS* user (JA *IQOS*) samples (page 24, lines 389-392).

**Reviewers 2 Major Comment 2.** We were confused by the individual TNP use prevalence values among the general population reported in Table 3. The overall prevalence for cigarettes ranges from 17.6, 17.3, and 16 % each year. If add the prevalence values for IQOS and e-cigarettes, results in total TNP prevalence of 20.1, 22.1, and 21.3% for each year. These values differ from the overall TNP prevalence of 18.5, 18.9, and 18.2 reported for each year in Table 2. Perhaps the authors can offer an explanation for this discrepancy.

***Authors’ Response Major Comment 2.*** We thank Reviewers 2 for this question on prevalence that often creates confusion when individual TNP prevalence values are shown that do not sum up to the overall TNP prevalence reported. Prevalence data for different tobacco product categories (e.g., cigarettes, HTPs, and e-cigarettes) can only be directly summed up to a higher-level use prevalence group (e.g., total prevalence of cigarette, HTP, and e-cigarette use) if each person only uses one category of tobacco product (e.g., only cigarettes, only HTPs, or only e-cigarettes). As soon as the same person uses more than one category (i.e., dual or poly use of different tobacco product categories), the person would be counted twice (dual use) or even more (poly use) in the higher-level prevalence group if the individual prevalence values would just be summed up, while correctly the person should only count once in the higher-level prevalence group (total prevalence of cigarette, HTP, and e-cigarette use).

**Reviewers 2 Major Comment 3.** While we agree that this evidence supports or does not contradict that switching to a smoke-free product like IQOS supports harm reduction, we suggest that the authors should be careful in drawing broader conclusions like “IQOS use behavior trends are in line with the principles of tobacco harm reduction”. Tobacco harm reduction cannot be viewed only in the narrow perspective of switching. An important consideration of unintended consequence is not only initiation among adults but also among youth. Additionally, as pointed in our comment above, the total TNP prevalence as calculated by us does not indicate a reduction in total tobacco product use, on the contrary, appears to increase over time. The authors should provide some context regarding these observations. Therefore, the authors should consider modifying the conclusions as “The use behavior trends suggest that IQOS has the potential to switch adult smokers from cigarettes to smoke-free products, which presents a harm reduction opportunity.” 

***Authors’ Response Major Comment 3.*** We thank Reviewers 2 for this comment. As suggested by Reviewers 2, we rephrased the conclusion section in the abstract (page 3) and discussion (page 24, lines 405-409) to make this point clearer. Regarding the overall TNP prevalence we reported, please see our response and explanations to Reviewers 2 Major Comment 2.

**Reviewers 2 Major Comment 4.** We caution the authors to avoid making causality inferences based on association. The authors should consider revising the conclusion - “The introduction of smoke-free TNPs does not lead to an unintended increase in overall TNP use in the general population, but rather drives a shift in TNP use patterns from cigarettes to smoke-free TNPs.” to reflect association rather than causation.

***Authors’ Response Major Comment 4.*** We agree with Reviewers 2 that because our study results are based on cross-sectional and descriptive data only, cause-effect inference cannot be drawn. We consider this limitation as a well-known fact inherent to cross-sectional studies. However, to make this point clearer, we amended the discussion (page 22, lines 305-308) and limitation section (page 24, lines 382-384) with regard to the limitations of the cross-sectional design.

***Reviewers 2 Major Comment 5.*** The survey lacks an appropriate characterization of quitting behavior among IQOS users. The survey does not present evidence regarding the likely interception of smokers who may have otherwise quit using TNP. If the survey did not assess this domain, the authors should cite this as a limitation. This further warrants a revision of the conclusions from not being so definitive but reflect the limitations of the survey results. 

***Authors’ Response Major Comment 5.*** We agree with Reviewers 2 that without addressing quitting behavior in our manuscript, we do not cover the information whether switching from cigarettes to *IQOS* may have prevented cigarette smokers from quitting all TNP. However, assessing quitting behavior was not in the scope of our manuscript. We still added the information that quitting behavior was not investigated to the limitation section of the discussion (page 24, lines 394-396).

**Reviewers 2 Major Comment 6.** The authors should include an explanation of the drop in response rates for JAIQOS samples from 19.4% in Y1 to 4.7% and 2.0% in Y2 and Y3. Given that the IQOS users changed significantly, from 350,000 to 6 million, the IQOS population likely changed over the course of the 3 surveys. The authors should consider accounting for the change in IQOS population by adjusting either during recruitment or during the data analysis.

***Authors’ Response Major Comment 6.*** The decline in response rates across the three study years observed for the JA *IQOS* samples can be explained by the fact that over time, the *IQOS* users increasingly received email invitations to various PMI surveys which might have generated a lower willingness to participate in our surveys.

 We included the explanation for the decreasing response rates related to the JA *IQOS *surveys in the discussion section (page 24, lines 374-377).

 As stated in the limitations section of the discussion (page 24, lines 386-389), because during the study years, we continuously monitored the response rates and compared them against pre-established quotas on the basis of age and sex, there was no need for further adjustment during recruitment or the data analysis because we still had sufficient samples (500 completed surveys) in each survey wave available.

**Reviewers 2 Major Comment 7.** The authors only present descriptive analyses of survey findings, however, they may want to consider including some trend analysis or other statistical testing to determine whether the changes over time are significant. Perhaps such an analysis may not yield robust findings from a three-year survey and authors may consider providing more statistical rigor in future reports.

***Authors’ Response Major Comment 7.*** We thank Reviewers 2 for this valid comment. Our statistical analysis plan for this 3-year study did only foresee descriptive analysis. For future studies, we are considering using more sophisticated trend analysis based on statistical methods.

**Reviewers 2 Major Comment 8.** We understand that the focus of this manuscript was among adults age >20 years. However, an important consideration is the impact of IQOS and other smoke-free products among youth, which is an important public health concern. The authors should mention that this manuscript only addresses adult use behavior and either briefly describe data for youth use based on published literature or national surveys. Some indication of initiation among different age groups of youth and young adults would have provided useful insights in the overall harm reduction potential of IQOS and other smoke-free products.

***Authors’ Response Major Comment 8.*** We agree with Reviewers 2 and amended the discussion (page 23, lines 363-365) and limitation section (page 24, lines 396-398), mentioning that our manuscript is based on Japanese adult participants only and that with regard to tobacco harm reduction, tobacco use prevalence among youth should be taken into consideration. The available literature suggests that tobacco initiation with I *QOS*/HTPs and prevalence of *IQOS*/HTPs among youth in Japan is low (pages 23-24, lines 365-373).

**Minor comments**

**Reviewers 2 Minor Comment 1.** The authors should consider including the supporting literature citations in the statement – “The existing standard TNP use questions are available in the literature.”.

***Authors’ Response Minor Comment 1.*** We thank the Reviewers 2 for this valuable comment. In the methods section, we included the links to the (i) CDC Adult Tobacco Use Questions of the National Health Interview Survey (NHIS), (ii) WHO/CDC/CPHA Questions of the Global Adult Tobacco Survey (GATS), (iii) NIH/FDA PATH Study questionnaires, and (iv) MHLW Tobacco Questions of the Japan National Health and Nutrition Survey (page 6, lines 107-112).

**Reviewers 2 Minor Comment 2.** The authors should provide a possible explanation of the discrepancy in the reported total HTP use prevalence of 11% by Hori et al (ref 34) which is almost twice of that reported in the current manuscript. This discrepancy cannot be solely explained by methodological differences.

***Authors’ Response Minor Comment 2.*** We believe that there are multiple conceptual and methodological differences that may have contributed to the lower overall HTP use prevalence (>5%) in our study than that observed by Hori et al. (11%). These differences between our study and the Hori et al. study, respectively, include i.a., (i) cross-sectional vs. longitudinal design, (ii) age ≥20 years vs. age range 15-69 years, (iii) no weighting methods vs. inverse probability weighting, (iv) no adjustments vs. multiple adjustment of the data, (v) definition of current use [at the time of the survey vs. past 30 days], and (vi) lifetime use criterion [100 tobacco sticks] vs. no lifetime use criterion to qualify as a HTP user.

 Of all these aspects, we believe that the difference in the age groups included in both studies (i.e., age ≥20 year in our study vs. 15-69 years in the Hori et al. study) had the largest impact on the difference in the observed overall HTP prevalence between our study and the study of Hori et al. In our study, we included also older age groups above 69 years, so the age distribution was skewed towards older age groups among whom, however, the *IQOS* use prevalence was very low with only about 1 %. We added this information to the discussion section (page 23, lines 327-337).

**Reviewers 2 Minor Comment 3.** In this manuscript, the IQOS sample could have been better characterized. Given that the authors had access to the IQOS user database, they could have followed transition behavior over time rather than indirectly comparing changes in average smoking prevalence against increase in IQOS user prevalence. Moreover, they may also report transitions between dual use to exclusive switching behavior. Perhaps in future reports, the authors can include more detailed analyses of transition behaviors.

***Authors’ Response Minor Comment 3.*** We thank reviewers 2 for this important comment. Because the present study provides only cross-sectional but not longitudinal data of the same participants follow-ed up over time, investigating transition behaviors was not intended in this study. However, in future studies, we will consider analyzing transition behaviors based on prospective/longitudinal data.

**Reviewers 2 Minor Comment 4.** We believe that the title for Table 6 should include JGAP not JPAG. Please correct the typo.

***Authors’ Response Minor Comment 4.*** We thank Reviewers 2 for spotting this typo. We have corrected it.
